# Syntheses, Characterization, Thermal, and Antimicrobial Studies of Lanthanum(III) Tolyl/Benzyldithiocarbonates

**DOI:** 10.1155/2014/780631

**Published:** 2014-04-09

**Authors:** Savit Andotra, Nidhi Kalgotra, Sushil K. Pandey

**Affiliations:** Department of Chemistry, University of Jammu, Baba Saheb Ambedkar Road, Jammu 180 006, India

## Abstract

Lanthanum(III) tris(*O*-tolyl/benzyldithiocarbonates), [La(ROCS_2_)] (R = *o*-, *m*-, *p*-CH_3_C_6_H_4_ and C_6_H_5_CH_2_), were isolated as yellow solid by the reaction of LaCl_3_ *·*7H_2_O with sodium salt of tolyl/benzyldithiocarbonates, ROCS_2_Na (R = *o*-, *m*-, *p*-CH_3_C_6_H_4_ and C_6_H_5_CH_2_), in methanol under anhydrous conditions in 1 : 3 molar ratio. These complexes have formed adducts with nitrogen and phosphorus donor molecules by straightforward reaction of these complexes with donor ligands, which have the composition of the type [La(ROCS_2_)_3_ *·*nL] (where n = 2, L = NC_5_H_5_ or P(C_6_H_5_)_3_ and n = 1, L = N_2_C_12_H_8_ or N_2_C_10_H_8_). Elemental analyses, mass, IR, TGA, and heteronuclear NMR (^1^H, ^13^C and ^31^P) spectroscopic studies indicated bidentate mode of bonding by dithiocarbonate ligands leading to hexacoordinated and octacoordinated geometry around the lanthanum atom. Antimicrobial (antifungal and antibacterial) activity of the free ligands and some of the complexes have also been investigated which exhibited significantly more activity for the complexes than the free ligands.

## 1. Introduction

Alkyldithiocarbonates, more commonly referred to as xanthates, were first prepared by Semeniuc et al. [1]. Their applications as vulcanizers [2], fungicides [3], and flotation agents [4, 5] in metallurgy have been described in the literature. The synthetic and structural chemistry of xanthates witnessed increased attention through the pioneering work of Winter [6], Tiekink and Winter [7], Hoskins and Pannan [8], and Dakternieks et al. [9]. Subsequently, extensive structural analyses were performed by Tiekink and Haiduc [10], which showed that these ligands can coordinate to metal atoms in a monodentate, isobidentate, or anisobidentate fashion. More recent applications of xanthates and other thio compounds are in the production of nanoparticles of metal sulphides [11, 12] and NLO properties [13, 14]. Metal xanthates are extensively used as pesticides [15], corrosion inhibitors [16], agricultural reagents [17], and quite recently in therapy for HIV infections [18]. Moreover, xanthates are also known to show antitumor properties [19, 20] and their antioxidant properties could be of importance for treating Alzheimer's disease [21]. These have extensively been used as intermediates in organic synthesis, in free radical polymerization, for rechargeable lithium ion batteries, and so forth [22, 23]. Sodium and potassium ethyl xanthate have antidotal effects on acute mercurial poisoning [24]. Much progress has recently been achieved in the coordination chemistry of lanthanides [25]. The design and synthesis of lanthanide(III) complexes with chelating ligands have many potential applications such as light-emitting devices, sensors, liquid crystalline materials, and chelate lasers [26]. Both lanthanum and xanthate find their applications in the field of medicinal chemistry [27, 28]. In spite of years of chemistry of the extensive and long term use of alkyl xanthates as ligands [29–35], structural and spectroscopic characterization have been rather limited with regard to the aryl xanthates [36, 37]. Fackler et al., however, reported the synthesis of thallium aryl xanthates which are in turn used for the metathetical synthesis of other metal derivatives [36]. We report herein for the first time the synthesis and characterization of* O*-tolyl/benzyl xanthates of lanthanum(III) and their adducts with nitrogen and phosphorus donor ligands like pyridine (NC_5_H_5_), triphenylphosphine [P(C_6_H_5_)_3_], 1,10-phenanthroline (N_2_C_12_H_8_), and 2,2′-bipyridyl (N_2_C_10_H_8_).

## 2. Experimental

### 2.1. Materials and Methods

Stringent precautions were taken to exclude moisture during the preparation of ligands. Moisture was carefully excluded throughout the experimental manipulations by using standard* Schlenk *techniques. Sodium salts of dithiocarbonates were obtained using literature procedures [2]. Toluene (Thomas Baker, B.P. 110°C) and* n*-hexane (Thomas Baker, B.P. 68-69°C) were freshly dried over sodium wire. Methanol (Thomas Baker, B.P. 64°C) was dried over P_2_O_5_ and CaCO_3_, respectively. Cresols (*ortho*-,* meta*-, and* para*-) and benzyl alcohol (Thomas Baker, B.P. 191°C, 203°C, 202°C, and 205°C) were purified by distillation prior to use.

### 2.2. Physical Measurements

Lanthanum was estimated gravimetrically as lanthanum oxide [38]. Elemental analyses (C, H, N, and S) were carried out on CHNS-932 Leco Elemental analyzer and ESI mass spectra of the compounds were recorded on ESQUIRE 3000–00037 spectrophotometer from Indian Institute of Integrative Medicine (IIIM), Jammu. The IR spectra were recorded in KBr pallets in the range of 4000–200 cm^−1^ on a Perkin Elmer spectrum RX1-FT IR spectrophotometer and multinuclear (^1^H, ^13^C, and ^31^P) NMR spectra were recorded in CDCl_3_ on a Brucker Avance II 400 MHz spectrometer using TMS as internal reference for ^1^H and ^13^C and 85% H_3_PO_4_ as external reference for ^31^P NMR at Sophisticated Analytical Instrumentation Facility (SAIF), Punjab University, Chandigarh. The thermogram was analyzed by using Perkin Elmer, diamond TG/DTA instrument. The thermogram was recorded in the temperature range from 30°C to 1000°C under nitrogen atmosphere from National Chemical Lab (NCL), Pune. Also the antifungal and antibacterial activity were tested under laboratory condition in the Bioassay Lab, Department of Chemistry, University of Jammu, Jammu, using classical poison food technique and agar well diffusion method.

### 2.3. Synthetic Procedures

#### 2.3.1. Synthesis of [La(*o*-CH_3_C_6_H_4_OCS_2_)_3_] (****5****)

A methanolic solution (~35 mL) of sodium* O*-(*o*-tolyl) dithiocarbonate (1.00 g, 4.84 mmol) was added to methanolic solution of lanthanum chloride (0.60 g, 1.61 mmol) with constant stiring at room temperature. Subsequently, the contents were refluxed for eight hours. The turbidity created by the byproduct (sodium chloride) was filtered off using alkoxy funnel fitted with G-4 sintered disc and volatiles were removed from the filtrate under reduced pressure. The solid thus obtained was extracted with chloroform (~20 cm^3^) by stirring overnight. Again the insoluble's were filtered off and the desired product [La(*o*-CH_3_C_6_H_4_OCS_2_)_3_] (**5**) was obtained from the filtrate as yellow solid.

The compounds** 6**–**8** reported herein were synthesized by using similar methodology and required stoichiometric weights. The relevant synthetic and analytical data are given in Table 1.

#### 2.3.2. Synthesis of [La(*o*-CH_3_C_6_H_4_OCS_2_)_3_·2NC_5_H_5_]** (**9**)**

Pyridine (0.11 g, 1.39 mmol) was added with constant stirring to a methanolic (~15 mL) solution of [La(*o*-CH_3_C_6_H_4_OCS_2_)_3_] (0.50 g, 0.72 mmol). The mixture was refluxed for two hours. The solvent was evaporated* in vacuo* and the product was washed with dry* n*-hexane for the sake of purity and finally dried under reduced pressure that resulted in the formation of the compound [La(*o*-CH_3_C_6_H_4_OCS_2_)_3_·2NC_5_H_5_] (**9**) in 89% yield.

The compounds** 10**–**24** reported herein were synthesized by using similar methodology and required stoichiometric weights. The relevant synthetic and analytical data are given in Table 1.

### 2.4. Antimicrobial Activity

#### 2.4.1. Antifungal Activity

Potato dextrose medium (PDA) was prepared in a flask and sterilized. Now, 100**μ**L of each sample was added to the PDA medium and poured into each sterilized petri plate. Mycelial discs taken from the standard culture (*Fusarium oxysporum*) of fungi were grown on PDA medium for 7 days. These cultures were used for aseptic inoculation in the sterilized petri dish. Standard cultures, inoculated at 28 ± 1°C, were used as the control. The efficiency of each sample was determined by measuring the radial fungal growth. The radial growth of the colony was measured in two directions at right angles to each other and the average of two replicates was recorded in each case. Data were expressed as percent inhibition over the control from the size of the colonies. The percent inhibition was calculated using the formula % Inhibition = ((*C* − *T*)/*C*)× 100, where *C* is the diameter of the fungus colony in the control plate after 96-hour incubation and *T* is the diameter of the fungus colony in the tested plate after the same incubation period.

#### 2.4.2. Antibacterial Activity

Test samples were prepared in different concentrations (250, 500, and 1000 ppm) in DMSO. Agar medium (20 mL) was poured into each petri plate. The plates were swabbed with broth cultures of the respective microorganisms* Klebsiella pneumonia* and* Bacillus cereus* and kept for 15 minutes for adsorption to take place. About 6 mm diameter holes were created in the seeded agar plates using a punch and 100 *μ*L of the DMSO solution of each test compound was poured into the wells. DMSO was used as the control for all the test compounds. After holding the plates at room temperature for 2 hrs to allow diffusion of the compounds into the agar then the plates were incubated at 37°C for 24 hrs. The antibacterial activity was determined by measuring the diameter of the inhibition zone. The entire tests were made in triplicates and the mean of the diameter of inhibition was calculated.

## 3. Results and Discussion

Reactions of lanthanum trichloride, LaCl_3_·7H_2_O, with sodium salt of (*o*-,* m*-, and* p*-tolyl/benzyl)dithiocarbonates, (*o*-,* m*-, and* p*-CH_3_C_6_H_4_OCS_2_)Na/(C_6_H_5_CH_2_OCS_2_)Na (**1–4**), in 1 : 3 molar ratio were carried out in methanol which resulted in the formation of complexes [La(ROCS_2_)_3_] (R =* o*-,* m*-,* p*-CH_3_C_6_H_4_ and C_6_H_5_CH_2_) (**5–8**) as yellow solid in 85–89% yield (1). Reactions of [(ROCS_2_)Na] with LaCl_3_·7H_2_O:

(1) $$\begin{array}{c} \underset{\left(1\text{–}4\right)}{3\text{ROC}{\text{S}}_{2}\text{Na}}+\text{LaC}{\text{l}}_{3}\cdot 7{\text{H}}_{2}\text{O}\;\underset{\vec{\;\text{–}3\text{NaCl},\;\;\text{Reflux}}}{\text{C}{\text{H}}_{3}\text{OH}}\;\underset{\left(5\text{–}8\right)}{\left[\text{La}{\left(\text{ROC}{\text{S}}_{2}\right)}_{3}\right]}\\ \left(\text{R}=o\text{-},\;\;m\text{-},\;\;p\text{-C}{\text{H}}_{3}{\text{C}}_{6}{\text{H}}_{4}\;\;\text{and}\;\;{\text{C}}_{6}{\text{H}}_{5}\text{C}{\text{H}}_{2}\right) \end{array}$$

The compounds of the type [La(ROCS_2_)_3_·nL] (**8–24**) (where n = 2, L = NC_5_H_5_ (**9–12**) or P(C_6_H_5_)_3_ (**13–16**) and n = 1, L = N_2_C_12_H_8_ (**17–20**) or N_2_C_10_H_8_ (**21–24**)) were synthesized by the addition reactions of lanthanum(III) tris(*O*-tolyl/benzyl dithiocarbonates) with nitrogen and phosphorus donor ligands in 1 : 2 or 1 : 1 molar ratio in methanol (2). The formation of these donor stabilized compounds indicates that tris-lanthanum complexes are lewis acids. These reactions were quite facile. Reactions of [La(ROCS_2_)_3_] with N and P donor ligands (n = 2, L = NC_5_H_5_ (**9**–**12**) or P(C_6_H_5_)_3_ (**13**–**16**) and n = 1, L = N_2_C_12_H_8_ (**17**–**20**) or N_2_C_10_H_8_ (**21**–**24**)):

(2) $$\begin{array}{c} \underset{\left(5\text{–}8\right)}{\left[\text{La}{\left(\text{ROC}{\text{S}}_{2}\right)}_{3}\right]}+\text{nL}\;\underset{\vec{\;\;\text{Reflux}\;}}{\text{C}{\text{H}}_{3}\text{OH}}\;\underset{\left(9\text{–}24\right)}{\left[\text{La}{\left(\text{ROC}{\text{S}}_{2}\right)}_{3}\cdot \text{nL}\right]}\\ \left(\text{R}=o\text{-},\;\;m\text{-},\;\;p\text{-C}{\text{H}}_{3}{\text{C}}_{6}{\text{H}}_{4}\;\;\text{and}\;\;{\text{C}}_{6}{\text{H}}_{5}\text{C}{\text{H}}_{2}\right) \end{array}$$

These compounds are soluble in ethanol, acetone, chloroform, and dichloromethane and insoluble in most hydrocarbon solvents. These compounds appear to be bit moisture sensitive; however, these can be kept unchanged under anhydrous atmosphere. These compounds are nonvolatile even under the reduced pressure and tend to decompose on heating. However, decomposition products could not be identified. The synthetic and analytical data are given in Table 1.

### 3.1. Spectroscopic Studies 

#### 3.1.1. Mass Spectra

The mass spectra of a few representative lanthanum(III) complexes and their adducts (**5**,** 9**,** 14**,** 19**,** 22**, and** 24**) have shown their molecular ion peak [M^+^] at* m/z* = 688, 846, 1213, 868, 844, and 844, respectively. The value of molecular ion peak [M]^+^ in these complexes is an indicative of monomeric nature. Some other peaks were also observed which corresponds to the fragmented species after the successive removal of different groups. Based on the presence of the peaks in the mass spectra of some of the representative complexes, the various fragments have been given in Table 2.

#### 3.1.2. IR Spectra

The characteristic stretching bands in the IR spectra (4000–200) cm^−1^were assigned by comparison with literature data [2, 14, 39, 40]. The IR spectra of these complexes exhibited band in the region 1260–1238 cm^−1^for *v*(C–O–C). The bands observed in the region 3059–3012 and 1601–1560 cm^−1^ were ascribed to ring vibrations in the cyclic dithiocarbonates. The presence of one strong band for *v*(C–S) in the region 1044–1034 cm^−1^ without a shoulder favors the bidentate linkage of the dithiocarbonate ligands with lanthanum atom. The presence of a new band ascribed to *v*(La–S) was present in the region 331–310 cm^−1^, which is indicative of formation of La–S bond in these complexes. The IR spectra of the adducts (**9**–**24**) have showed all the bands observed in the parent lanthanum-dithiocarbonates and bands characteristic of donor ligands (NC_5_H_5_, P(C_6_H_5_)_3_, N_2_C_12_H_8_ and N_2_C_10_H_8_) in the regions 455–442 and 402–399 cm^−1^, which may be assigned to *v*(La–N) and *v*(La–P) bonding modes, respectively. The IR spectral values of the complexes are given in Table 3.

#### 3.1.3. ^1^HNMR Spectra

In ^1^H NMR spectra, the signals for the –CH_3_ (tolyl ring) and –CH_2_ (benzyl ring) protons were observed at 2.22–2.33 and 4.50–4.61 ppm as singlet. The protons of the C_6_H_4_ (tolyl) and C_6_H_5_ (benzyl ring) gave signals in the range 6.22–7.23 and 7.10–7.64 ppm as multiplets. This chemical shift has no deviation either to lower or higher field side compared to the parent ligands. There were two resonances for the ring protons of* para* complexes whereas four resonances were observed for* ortho-* and* meta*derivatives.^ 1^H NMR spectra of the addition complexes exhibited the characteristic proton signals of the tris(*o-, m-,* and* p-*tolyl/benzyldithicarbonate)lanthanum(III) complexes along with the chemical shifts for aromatic protons for the donor ligands. The chemical shifts for aromatic protons of triphenylphosphine moiety in the complexes** 13**–**16** were observed in the region 7.22–7.61 ppm as multiplet. In case of adducts with nitrogen donor ligand, the chemical shift for aromatic protons of pyridine in the complexes** 9**–**12** was observedin the region 7.60–8.41 ppm as multiplet. The complexes** 17**–**20** have shown the characteristic resonances for phenyl protons of 1,10-phenanthroline at 7.23–8.91 ppm. The chemical shift for aryl protons of the bipyridine appeared in the region 7.10–8.59 ppm for complexes** 21**–**24**. The presence of all characteristic chemical shifts in the ^1^H NMR spectra favors the formation of these complexes. The ^1^H NMR spectral data of these complexes are given in Table 4.

#### 3.1.4. ^31^P NMR Spectra

^31^P NMR spectra of the addition complexes (**13–16**) exhibited the signal for the phosphorus atom of the triphenylphosphine moiety as a singlet at −4.52 to −5.32 ppm. The ^31^P NMR resonances of bound ligand are shifted to downfield compared with those of the free triphenylphosphine. The relevant ^31^P NMR spectral data of these complexes are given in the Table 4.

#### 3.1.5. ^13^C NMR Spectra

^13^C NMR spectra of few representative complexes (**6-7**,** 11-12**,** 14-15**,** 17-18**,** 21**, and** 24**) have shown the appearance of the chemical shift for all the carbon nuclei in their characteristic region. The chemical shift for methyl (–CH_3_) and methylene (–CH_2_) carbon occurred in the range 19.63–21.10 and 71.02–71.23 ppm, respectively. The carbon nuclei of phenyl groups (–C_6_H_5_ and –C_6_H_4_) have displayed their resonance in the region 112.21–130.02 ppm. The carbon attached to the methyl and methylene substituted carbon of the phenyl ring in the respective compounds appeared at 123.51–139.50 and 137.41–138.03 ppm, respectively. The peak in the region 152.10–158.90 ppm was due to the carbon attached to the oxygen in the tolyl derivatives. The chemical shift for the dithiocarbonate carbon (–(O)CS_2_) appeared at 164.00–169.16 ppm. The ^13^C NMR spectra of the addition complexes exhibited the signals of the carbon nucleus of the donor moieties in addition to the characteristic chemical shifts indicated above. The aryl carbon nuclei of the pyridine (**11**-**12**) and triphenylphosphine (**14**-**15**) resonated at 118.71–148.02 ppm and 127.02–138.01 ppm, respectively. The aryl carbon nuclei of the phenanthroline (**17**-**18**) and bipyridine (**21**–**24**) gave their resonance at 120.02–150.00 ppm and 120.12–153.80 ppm, respectively. The ^13^C NMR spectral data of the complexes are given in the Table 5.

#### 3.1.6. Thermogravimetric Analysis

**Figure 1:**
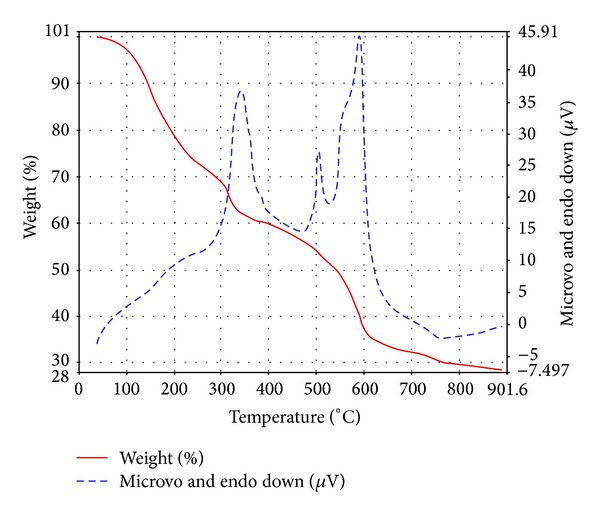
TGA curve of the complex [La(*p*-CH_3_C_6_H_4_OCS_2_)_3_] (**5**).

The thermal properties of the complexes were studied by TGA in the temperature ranging from 30–1000°C under nitrogen atmosphere. The content of a particular component in a complex changes with its composition and structure. These can be determined based on mass losses of these components in the thermogravimetric plots of the complexes. The thermogravimetric analysis of the complex, [La(*p*-CH_3_C_6_H_4_OCS_2_)_3_] (**5**) displayed a thermolysis step that covers a temperature range from 150 to 900°C. The thermogram (Figure 1) exhibited the decline curve characteristic for dithiocarbonate complexes. The diagnostic weight loss of initial weight occurs in the steeply descending segment of the TGA curve. This weight loss, that is, 27.5% at 243.4°C, is due to the decomposition of the dithiocarbonate corresponding to [La(*p*-CH_3_C_6_H_4_OCS_2_)_2_], (the calculated weight loss is 26.7%) as an intermediate product, which agrees with thermogravimetric data for dithiocarbonates. Another important weight loss 47.3% (obs.) occurs at 558.5°C temperature corresponding to the formation of [La(OCS_2_)_2_] (weight loss calculated 47.0%). The decomposition continues to about 800°C at which most of the organic part of the compound has been lost. This sharp decomposition period brings about 68–71% weight loss in the lanthanum complex and led to the complete formation of metal sulfide, that is, LaS_2_ (weight loss calculated 70.5%, observed 70.6%), at 813°C. The calculated mass change agrees favorably with experimental values.

#### 3.1.7. Antimicrobial Activity

**Figure 2:**
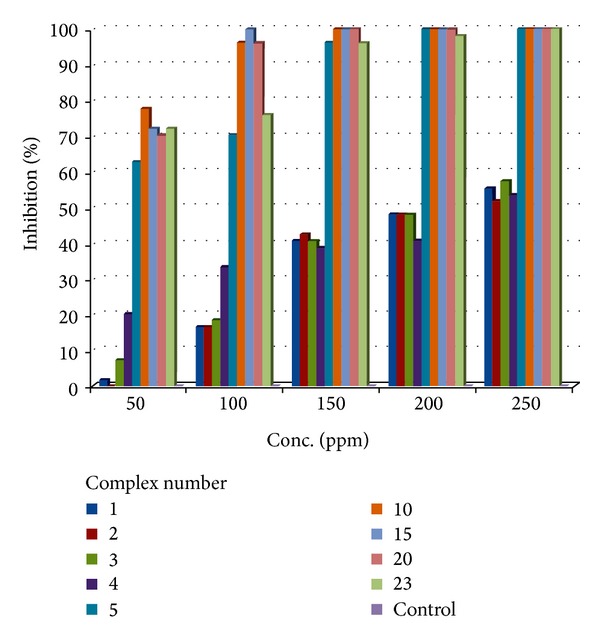
Comparison of antifungal activity of the ligands and their lanthanum(III) complexes.

*(1)   Antibacterial to Antifungal.* The* in vitro* biological screening effects of all the ligands and some of the complexes (**5**,** 10**,** 15**,** 20**, and** 23**) were tested against the fungus* Fusarium oxysporum* f. sp. Capsici causing vascular wilt of chilli. The antifungal screening data are given in Table 6, which shows that complexes have higher activity than free ligands. The colony diameter of the fungus decreases on enhancing the concentration of the complex; that is, all the complexes inhibited the growth of fungus significantly. This shows a linear relationship between concentration and percent inhibition. The increase in antifungal activity may be attributed to faster diffusion of metal complexes as a whole through the cell membrane or due to combined activity effect of the metal and the ligand. It is also evident from the antifungal screening data that adducts of nitrogen and phosphorous donor ligands are more potent than the parent complex. The chelation theory accounts for the increased activity of the metal complexes [41]. On chelating, the polarity of the metal ion will be reduced to a greater extent due to overlap of the ligand orbital and the partial sharing of the positive charge of the metal ion with donor group. The comparison of antifungal activity of all the ligands and some of the complexes is described diagrammatically in Figure 2.

*(2) Antibacterial Activity.* Antibacterial* in vitro* studies against two bacterial strains involve Gram-negative* Klebsiella pneumonia* and* Gram-positive* Bacillus cereus using penicillin as standard antibacterial drug. Antibacterial screening data are given in Table 7. These studies revealed that free ligands are inactive against the bacterial strains but metal complexes shows higher activity than free ligands but lower activity than reference drug that is, penicillin. However, the complex [La(C_6_H_4_CH_2_OCS_2_)_3_·N_2_C_12_H_8_] (**20**) shows pronounced activity against* Klebsiella pneumonia* and* Bacillus cereus* even more than reference drug.

## 4. Conclusions

**Figure 3:**
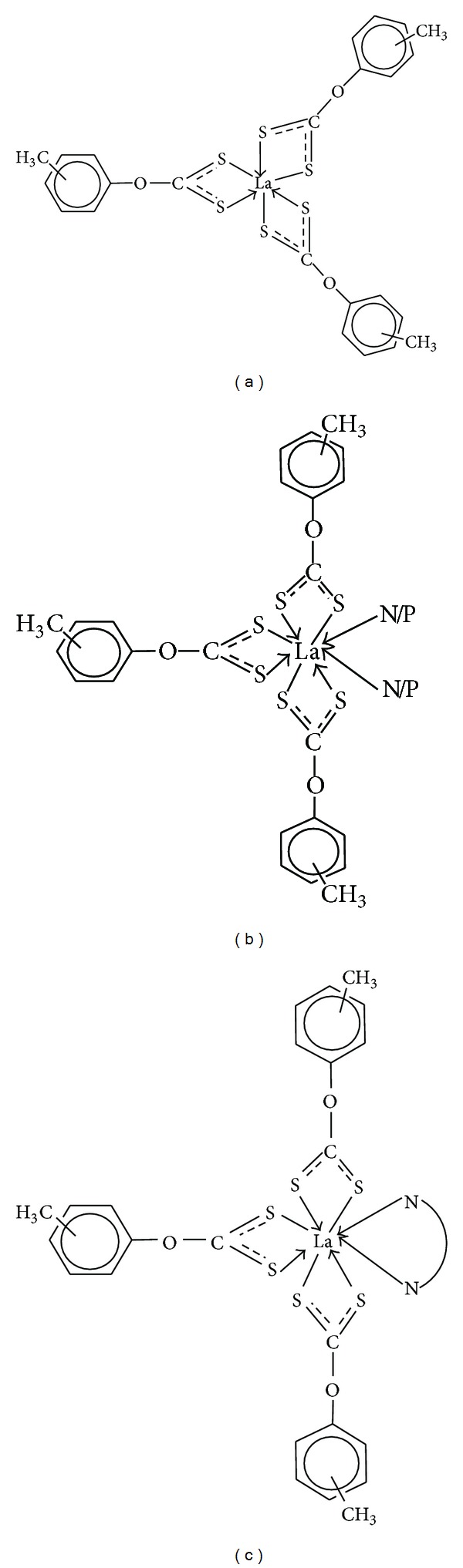
(a) Proposed hexacoordinate structure for [La(*o-, m-* and* p-*CH_3_C_6_H_4_OCS_2_)_3_] (**5**–**7**). (b) Proposed octacoordinate structure for [La(*o-, m-* and* p-*CH_3_C_6_H_4_OCS_2_)_3_.2N/P] (**9**–**11**,** 13**–**15**) [N = NC_5_H_5_ (**9**–**12**) and P = P(C_6_H_5_)_3_ (**13**–**15**)]. (c) Proposed octacoordinate structure for [La(*o-, m-* and* p-*CH_3_C_6_H_4_OCS_2_)_3_·N_2_C_12_H_8_/N_2_C_10_H_8_] [(**17**–**19**,** 21**–**23**)].

On the basis of elemental analysis, mass, IR, and NMR (^1^H, ^13^C and ^31^P NMR) spectral studies and in conjunction with the literature reports [39, 42–45], a hexacoordinate structure may be proposed for lanthanum(III)tris(*O-*tolyl/benzyldithiocarbonates) (**5**–**8**) in Figure 3(a) and octacoordinate structure may be proposed for adducts of lanthanum(III)tris(*O*-tolyl/benyldithiocarbonates) (**9**–**24**) in which tolyldithiocarbonate ligands behaved in bidentate manner. Hence, the lanthanum atom is coordinated by six sulfur atoms of the dithiocarbonate and two nitrogen atoms of the two pyridine molecules in the compounds** 9**–**12** as shown in Figure 3(b). In the compounds** 13**–**16** lanthanum atom is coordinated with the two phosphorus atoms of the two triphenylphosphine molecules and six sulfur atoms of the dithiocarbonate Figure 3(b). The octacoordination by lanthanum in the compounds** 17**–**24** is achieved by coordination with six sulfur atoms of dithiocarbonate ligand and two nitrogen atoms of phenanthroline and bipyridyl molecule as described in Figure 3(c). The benzyl analogues (**8**,** 12**,** 16**,** 20**, and** 24**) have similar structures.

## Figures and Tables

**Table 1:** Synthetic and analytical data of tolyl/benzyl dithiocarbonates of La(III) and their adducts.

S. number	Reactants *g* (mmol)		Molar ratio	Reflux time (hrs.)	Product (Physical state)	M.P (°C) (dec.)	Yield %	Analysis % found (Calcd.)				
	ROCS_2_Na/[(ROCS_2_)_3_La]	LaCl_3_/L*						La	C	H	N	S
**5.**	1.00 (4.84)	0.60 (1.61)	3 : 1	8	[(*o*-CH_3_C_6_H_4_OCS_2_)_3_La] (yellow solid)	120	85	20.15 (20.17)	41.81 (41.85)	3.05 (3.07)	—	27.90 (27.93)
**6.**	1.00 (4.84)	0.60 (1.61)	3 : 1	8	[(*m*-CH_3_C_6_H_4_OCS_2_)_3_La] (yellow solid)	117	89	20.14 (20.17)	41.80 (41.85)	3.04 (3.07)	—	27.90 (27.93)
**7.**	1.00 (4.84)	0.60 (1.61)	3 : 1	8	[(*p*-CH_3_C_6_H_4_OCS_2_)_3_La] (yellow solid)	118	87	20.15 (20.17)	41.82 (41.85)	3.05 (3.07)	—	27.89 (27.93)
**8.**	1.00 (4.84)	0.60 (1.61)	3 : 1	8	[(C_6_H_5_CH_2_OCS_2_)_3_La] (yellow solid)	121	86	20.13 (20.17)	41.81 (41.85)	3.03 (3.07)	—	27.88 (27.93)
**9.**	0.50 (0.72)	0.11 (1.39)	1 : 2	2	[(*o*-CH_3_C_6_H_4_OCS_2_)_3_La·2(NC_5_H_5_)] (Yellow solid)	130	89	16.36 (16.40)	48.20 (48.22)	3.66 (3.69)	3.29 (3.31)	22.70 (22.72)
**10.**	0.50 (0.72)	0.11 (1.39)	1 : 2	2	[(*m*-CH_3_C_6_H_4_OCS_2_)_3_La·2(NC_5_H_5_)] (Yellow solid)	137	86	16.35 (16.40)	48.20 (48.22)	3.58 (3.69)	3.30 (3.31)	22.69 (22.72)
**11.**	0.50 (0.72)	0.11 (1.39)	1 : 2	2	[(*p*-CH_3_C_6_H_4_OCS_2_)_3_La·2(NC_5_H_5_)] (Yellow solid)	134	88	16.37 (16.40)	48.19 (48.22)	3.67 (3.69)	3.29 (3.31)	22.71 (22.72)
**12.**	0.50 (0.72)	0.11 (1.39)	1 : 2	2	[(C_6_H_5_CH_2_OCS_2_)_3_La·2(NC_5_H_5_)] (Yellow solid)	138	87	16.37 (16.40)	48.18 (48.22)	3.66 (3.69)	3.28 (3.31)	22.71 (22.72)
**13.**	0.50 (0.72)	0.38 (1.42)	1 : 2	5	[(*o*-CH_3_C_6_H_4_OCS_2_)_3_La·2P(C_6_H_5_)_3_] (Pale yellow solid)	145	88	11.43 (11.45)	59.38 (59.40)	4.20 (4.24)	—	15.85 (15.86)
**14.**	0.50 (0.72)	0.38 (1.42)	1 : 2	5	[(*m*-CH_3_C_6_H_4_OCS_2_)_3_La·2P(C_6_H_5_)_3_] (Pale yellow solid)	148	85	11.42 (11.45)	59.36 (59.40)	4.20 (4.24)	—	15.87 (15.86)
**15.**	0.50 (0.72)	0.38 (1.42)	1 : 2	5	[(*p*-CH_3_C_6_H_4_CH_2_OCS_2_)_3_ La·2P(C_6_H_5_)_3_] (Pale yellow solid)	146	86	11.43 (11.45)	59.36 (59.40)	4.23 (4.24)	—	15.84 (15.86)
**16.**	0.50 (0.72)	0.38 (1.42)	1 : 2	5	[(C_6_H_5_CH_2_OCS_2_)_3_La·2P(C_6_H_5_)_3_] (Pale yellow solid)	150	88	11.42 (11.45)	59.37 (59.40)	4.20 (4.24)	—	15.84 (15.86)
**17.**	0.50 (0.72)	0.13 (0.72)	1 : 1	5	[(*o*-CH_3_C_6_H_4_OCS_2_)_3_La·N_2_C_12_H_8_] (Yellowish brown solid)	160	89	15.95 (15.99)	49.72 (49.76)	3.32 (3.36)	3.19 (3.22)	22.10 (22.14)
**18.**	0.50 (0.72)	0.13 (0.72)	1 : 1	5	[(*m*-CH_3_C_6_H_4_OCS_2_)_3_La·N_2_C_12_H_8_] (Yellowish brown solid)	155	87	15.96 (15.99)	49.73 (49.76)	3.33 (3.36)	3.18 (3.22)	22.11 (22.14)
**19.**	0.50 (0.72)	0.13 (0.72)	1 : 1	5	[(*p*-CH_3_C_6_H_4_OCS_2_)_3_La·N_2_C_12_H_8_] (Yellowish brown solid)	158	88	15.95 (15.99)	49.73 (49.76)	3.33 (3.36)	3.18 (3.22)	22.11 (22.14)
**20.**	0.50 (0.72)	0.13 (0.72)	1 : 1	5	[(C_6_H_5_CH_2_OCS_2_)_3_La·N_2_C_12_H_8_] (Yellowish brown solid)	157	88	15.97 (15.99)	49.74 (49.76)	3.32 (3.36)	3.19 (3.22)	22.11 (22.14)
**21.**	0.50 (0.72)	0.11 (0.70)	1 : 1	5	[(*o*-CH_3_C_6_H_4_OCS_2_)_3_La·N_2_C_10_H_8_] (Orange solid)	162	85	16.40 (16.44)	48.32 (48.33)	3.43 (3.46)	3.28 (3.32)	22.75 (22.77)
**22.**	0.50 (0.72)	0.11 (0.70)	1 : 1	5	[(*m*-CH_3_C_6_H_4_OCS_2_)_3_La·N_2_C_10_H_8_] (Orange-yellow solid)	165	88	16.40 (16.44)	48.29 (48.33)	3.44 (3.46)	3.29 (3.32)	2.75 (22.77)
**23.**	0.50 (0.72)	0.11 (0.70)	1 : 1	5	[(*p*-CH_3_C_6_H_4_OCS_2_)_3_La·N_2_C_10_H_8_] (Orange yellow solid)	164	87	16.41 (16.44)	48.30 (48.33)	3.43 (3.46)	3.30 (3.32)	22.74 (22.77)
**24.**	0.50 (0.72)	0.11 (0.70)	1 : 1	5	[(C_6_H_5_CH_2_OCS_2_)_3_La·N_2_C_10_H_8_] (Orange solid)	162	89	16.42 (16.44)	48.30 (48.33)	3.42 (3.46)	3.29 (3.32)	22.74 (22.77)

R = *o* − , *m* − , *p*−CH_3_C_6_H_4_ and C_6_H_5_CH_2_L* = NC_5_H_5_ (**9–12**) or P(C_6_H_5_)_3 _(**13–16**) and for *n* = 1, L* = N_2_C_12_H_8_ (**17–20**) or N_2_C_10_H_8_ (**21–24**).

**Table 2:** Mass spectral data of tolyl/benzyl dithiocarbonates of La(III) and their adducts.

S. No.*	M.W.	*m*/*z*, Relative intensities of the ions and assignment
**5.**	688	[M^+^] 688 (11) [La(*o*-CH_3_C_6_H_4_OCS_2_)_3_]; [M^+^] 183 (30) [*o*-CH_3_C_6_H_4_OCS_2_]; [M^+^] 107 (18) [*o*-CH_3_C_6_H_4_O]; [M^+^] 504 (15) [La(*o*-CH_3_C_6_H_4_OCS_2_)_2_]; [M^+^] 321 (7) [La(*o*-CH_3_C_6_H_4_OCS_2_)]; [M^+^] 503 (15) [(C_6_H_4_OCS_2_)_3_]; [M^+^] 474 (9) [La(C_6_H_4_OCS_2_)_2_]; [M^+^] 306 (10) [La(C_6_H_4_OCS_2_)]; [M^+^] 168 (8) [(C_6_H_4_OCS_2_)].
**9.**	846	[M^−^] 846 (7) [La(*o-*CH_3_C_6_H_4_OCS_2_)_3_La.2NC_5_H_5_]; [M^+^] 687 (15) [La(*o-*CH_3_C_6_H_4_OCS_2_)_3_]; [M^−^] 183 (15) [(*o-*CH_3_C_6_H_4_OCS_2_)]; [M^−^] 107 (16) [*o-*CH_3_C_6_H_4_O]; [M^+^] 662 (15) [La(*o-*CH_3_C_6_H_4_OCS_2_)_2_·2NC_5_H_5_]; [M^+^] 296 (12) [La.2NC_5_H_5_].
**14.**	1213	[M^+^] 1213 (5) [La(*m-*CH_3_C_6_H_4_OCS_2_)_3_·2P(C_6_H_5_)_3_]; [M^+^] 949 (6) [La(*m-*CH_3_C_6_H_4_OCS_2_)_3_·P(C_6_H_5_)_3_]; [M^+^] 687 (4) [La(*m-*CH_3_C_6_H_4_OCS_2_)_3_]; [M^+^] 1029 (9) [La(*m-*CH_3_C_6_H_4_OCS_2_)_2_·2P(C_6_H_5_)_3_]; [M^+^] 504 (6) [La(*m-*CH_3_C_6_H_4_OCS_2_)_2_]; [M^+^] 183 (7) [*m-*CH_3_C_6_H_4_OCS_2_]; [M^−^] 107 (7) [*m-*CH_3_C_6_H_4_O].
**19.**	868	[M^+^] 868 (6) [La(*p-*CH_3_C_6_H_4_OCS_2_)_3_·N_2_C_12_H_8_]; [M^+^] 687 (8) [La(*p-*CH_3_C_6_H_4_OCS_2_)_3_]; [M^+^] 684 (7) [La(*p-*CH_3_C_6_H_4_OCS_2_)_2_·N_2_C_12_H_8_]; [M^+^] 321 (4) [La(*p-*CH_3_C_6_H_4_OCS_2_)]; [M^−^] 107 (6) [*p-*CH_3_C_6_H_4_O]; [M^+^] 318 (5) [La·N_2_C_12_H_8_]; [M^+^] 180 (5) [N_2_C_12_H_8_];
**22.**	844	[M^+^] 844 (6) [La(*m-*CH_3_C_6_H_4_OCS_2_)_3_·N_2_C_10_H_8_]; [M^+^] 687 (8) [La(*m-*CH_3_C_6_H_4_OCS_2_)_3_]; [M^+^] 687 (8) [La(*m-*CH_3_C_6_H_4_OCS_2_)_2_]; [M^+^] 183 (5) [*o-*CH_3_C_6_H_4_OCS_2_]; [M^−^] 477 (6) [La(*m-*CH_3_C_6_H_4_OCS_2_)·N_2_C_10_H_8_]; [M^+^] 294 (4) [La·N_2_C_10_H_8_]; [M^+^] 156 (20) [N_2_C_10_H_8_].
**24.**	844	[M^+^] 844 (10) [La(C_6_H_5_CH_2_OCS_2_)_3_·N_2_C_10_H_8_]; [M^+^] 687 (6) [La(C_6_H_5_CH_2_OCS_2_)_3_]; [M^+^] 613 (4) [La(CH_2_OCS_2_)_3_·N_2_C_10_H_8_)][M^+^] 456 (13) [La(CH_2_OCS_2_)_3_]; [M^+^] 294 (6) [La·N_2_C_10_H_8_]; [M^+^] 156 (14) [N_2_C_10_H_8_].

Bracket = *m*/*z*; parentheses = intensities in %; *S. number of the complexes is according to Table 1.

**Table 3:** IR spectral data of tolyl/benzyl dithiocarbonates of La(III) and their adducts (cm^−1^).

S. number*	*v*C–O–C	*v*C–S	Aromatic stretching		*v*La–S	*v*La–N/La–P**
			*v*C–H	*v*C–C		
**5.**	1248, s	1039, m	3018, b	1599, s	312, w	—
**6.**	1249, s	1036, m	3012, b	1595, s	320, w	—
**7.**	1248, s	1038, m	3028, b	1596, s	325, w	—
**8.**	1238, s	1037, m	3029, b	1594, s	310, w	—
**9.**	1239, s	1039, m	3030, b	1595, s	330, w	450, w
**10.**	1245, s	1035, m	3055, b	1598, s	324, w	455, w
**11.**	1239, s	1040, m	3047, b	1597, s	320, w	445, w
**12.**	1247, s	1034, m	3057, b	1590, s	325, w	448, w
**13.**	1241, s	1039, m	3044, b	1596, s	320, w	400, w
**14.**	1248,s	1037, m	3041, b	1598, s	328, w	402, w
**15.**	1255, s	1034, m	3012, b	1560, s	328, w	399, w
**16.**	1260, s	1040, m	3045, b	1601, s	325, w	401, w
**17.**	1254, s	1044, m	3052, b	1593, s	330, w	450, w
**18.**	1248, s	1042, m	3024, b	1594, s	331, w	452, w
**19.**	1249, s	1042, m	3049, b	1598, s	326, w	442, w
**20.**	1238, s	1038, m	3059, b	1597, s	328, w	447, w
**21.**	1244, s	1040, m	3044, b	1599, s	325, w	445, w
**22.**	1239, s	1041, m	3028, b	1598, s	320, w	446, w
**23.**	1240, s	1038, m	3044, b	1595, s	315, w	451, w
**24.**	1248, s	1039, m	3045, b	1598, s	312, w	450, w

s: sharp, b: broad, m: medium, and w: weak; **vLa-N for complexes **9**–**12** and **17**–**24** and *v*La-P for complexes **13**–**16.**

*S. number of the complexes is according to Table 1.

**Table 4:** ^
1^H and ^31^P NMR spectral data of tolyl/benzyl dithiocarbonates of La(III) and their adducts in CDCl_3_ (in ppm).

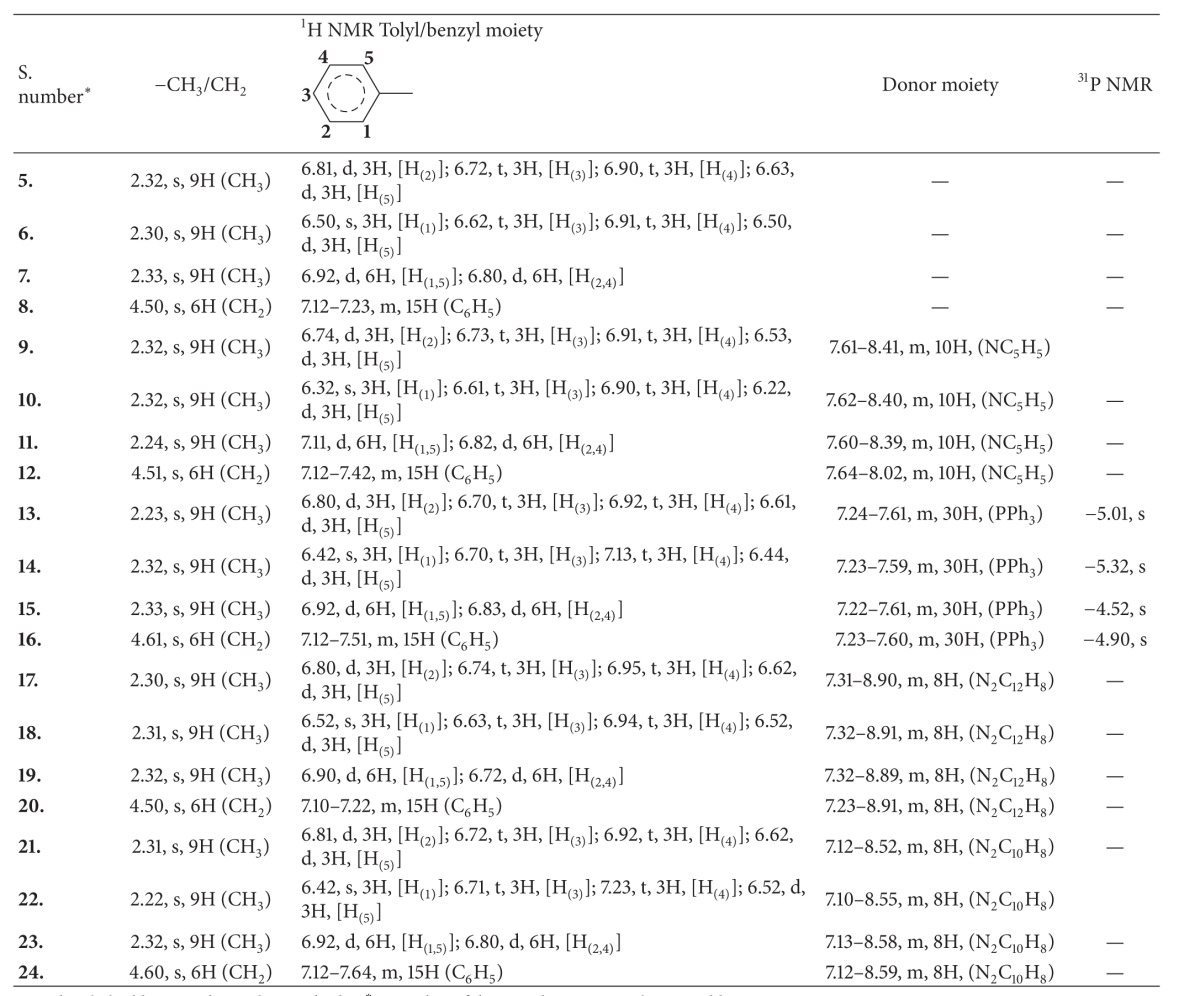

s: singlet, d: doublet, t: triplet, and m: multiplet; *S. number of the complexes is according to Table 1.

**Table 5:** ^
13^C NMR spectral data of tolyl/benzyl dithiocarbonates of La(III) and their adducts in CDCl_3_ (in ppm).

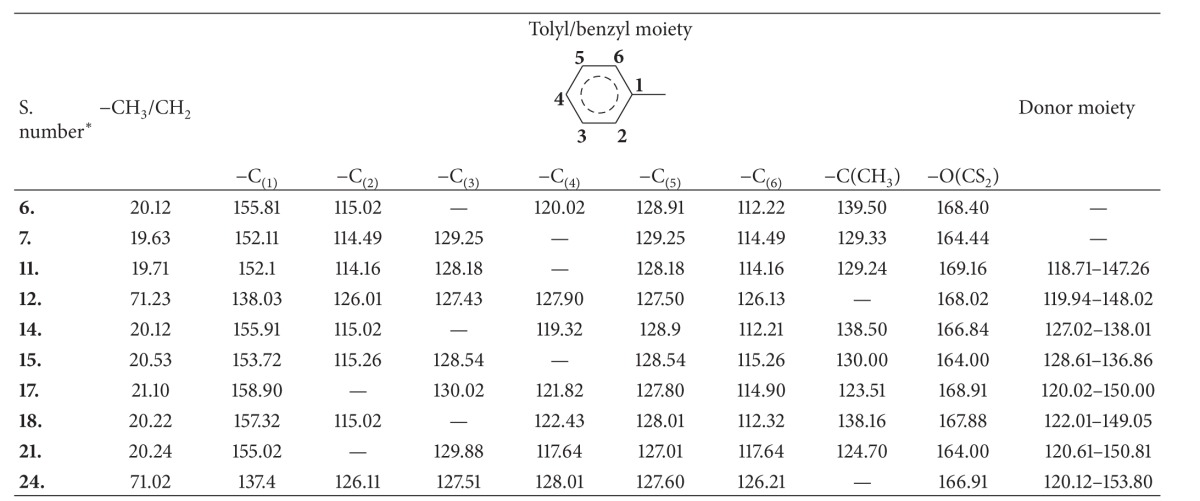

**Table 6:** *In vitro* evaluation of the ligands and their lanthanum(III) complexes against the fungus *Fusarium oxysporum* f. sp..

S. number*	Concentration. (ppm)	Colony diameter (cm)	Inhibition over control (%)	Concentration. (ppm)	Colony diameter (cm)	Inhibition over control (%)	Concentration. (ppm)	Colony diameter (cm)	Inhibition over control (%)	Concentration. (ppm)	Colony diameter (cm)	Inhibition over control (%)	Concentration. (ppm)	Colony diameter (cm)	Inhibition over control (%)
**1.**	**50**	5.3	1.8	**100**	4.5	16.6	**150**	3.2	40.7	**200**	2.8	48.1	**250**	2.4	55.5
**2.**	**50**	5.4	0	**100**	4.5	16.6	**150**	3.1	42.5	**200**	2.8	48.1	**250**	2.6	51.8
**3.**	**50**	5	7.4	**100**	4.4	18.5	**150**	3.2	40.7	**200**	2.8	48.1	**250**	2.3	57.4
**4.**	**50**	4.3	20.3	**100**	3.6	33.3	**150**	3.3	38.8	**200**	3.2	40.7	**250**	2.5	53.7
**5.**	**50**	2	62.9	**100**	1.6	70.3	**150**	0.2	96.2	**200**	0	100	**250**	0	100
**10.**	**50**	1.5	77.7	**100**	0.2	96.2	**150**	0	100	**200**	0	100	**250**	0	100
**15.**	**50**	1.5	72.2	**100**	0	100	**150**	0	100	**200**	0	100	**250**	0	100
**20.**	**50**	1.6	70.3	**100**	0.2	96.2	**150**	0	100	**200**	0	100	**250**	0	100
**23.**	**50**	1.5	72.2	**100**	1.3	75.9	**150**	0.2	96.2	**200**	0.1	98.1	**250**	0	100
**Control**	**50**	5.4	0	**100**	5.4	0	**150**	5.4	0	**200**	5.4	0	**250**	5.4	0

Inhibition over control (%) = [(C−T)/C] × 100, where C is the mean control (colony diameter) and T is the treatment (colony diameter).

*S. number of the complexes is according to Table 1.

**Table 7:** Antibacterial screening of the ligands and their lanthanum(III) complexes.

S. number*	Diameter of inhibition zone (cm) (conc. in ppm)					
	*Klebsiella pneumonia *(−)			*Bacillus cereus *(+)		
	250 ppm	500 ppm	1000 ppm	250 ppm	500 ppm	1000 ppm
**1.**	N.I	N.I	N.I	N.I	N.I	N.I
**2.**	N.I	N.I	N.I	N.I	N.I	N.I
**3.**	N.I	N.I	N.I	N.I	N.I	N.I
**4.**	N.I	N.I	N.I	N.I	N.I	N.I
**5.**	1.3	1.9	2.2	0.9	1.3	1.4
**10.**	1.6	2.5	2.7	2.2	2.7	3.2
**15.**	2.2	2.8	3.2	0.8	1.7	2.2
**20.**	2.4	3.5	3.6	2.8	3.4	3.6
**23.**	0.6	2.1	2.3	1.6	2.6	2.9
**Penicillin.**	2.2	2.6	2.9	2.4	2.8	2.9

N.I: no inhibition; *S. number of the complexes is according to Table 1.
